# Supplementary material for: GeDi: simplifying gene set distances for enhanced omics interpretation in R/Bioconductor
Source: BMC Bioinformatics. 2025 Dec 7;27:14. doi: 10.1186/s12859-025-06335-6 (PMC12809992; doi:10.1186/s12859-025-06335-6)
Supplement: Supplementary file 1 — Supplementary Material 1. [file 12859_2025_6335_MOESM1_ESM.zip › Supplementary_Material/Supplementary_Figure_1.pdf]

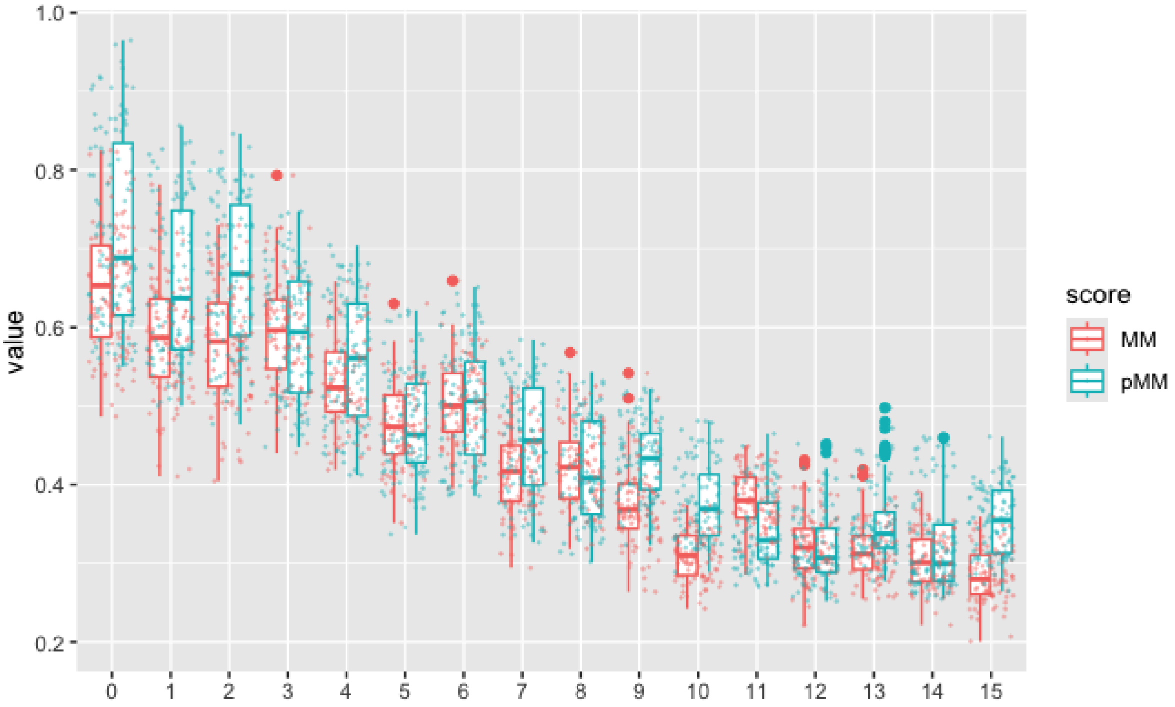

Supplementary Figure 1: The figure shows the box plot comparison of the ARI scores of the Louvain clustering results based on the MM and pMM distance scores. The x-axis shows the number of genes removed from the gene sets, while the y-axis shows the value of the ARI score.
